# Genetic and Morpho-Physiological Differences among Transgenic and No-Transgenic Cotton Cultivars

**DOI:** 10.3390/plants12193437

**Published:** 2023-09-29

**Authors:** Li Liu, Dan Wang, Jinping Hua, Xianhui Kong, Xuwen Wang, Juan Wang, Aijun Si, Fuxiang Zhao, Wenhao Liu, Yu Yu, Zhiwen Chen

**Affiliations:** 1 Cotton Institute, Xinjiang Academy of Agricultural and Reclamation Science/Northwest Inland Region Key Laboratory of Cotton Biology and Genetic Breeding, Shihezi 832000, China; cottonliuli@sina.com (L.L.); kxh920@sohu.com (X.K.); wxw629@163.com (X.W.); cottonwj@126.com (J.W.); siaijun1002@163.com (A.S.); 1365100845@163.com (F.Z.); whylwh2016@163.com (W.L.); 2 Laboratory of Cotton Genetics, Genomics and Breeding/Beijing Key Laboratory of Crop Genetic Improvement/Key Laboratory of Crop Heterosis and Utilization of Ministry of Education, College of Agronomy and Biotechnology, China Agricultural University, Beijing 100193, China; wangd@163.com (D.W.); jinping_hua@cau.edu.cn (J.H.); 3 Key Laboratory of Graphene Forestry Application of National Forest and Grass Administration, Engineering Research Center of Coal-Based Ecological Carbon Sequestration Technology of the Ministry of Education, Shanxi Datong University, Datong 037009, China

**Keywords:** fatty acids, fiber development, gene expression, seed oil content, upland cotton (*Gossypium hirsutum*)

## Abstract

Three carbon-chain extension genes associated with fatty acid synthesis in upland cotton (*Gossypium hirsutum*), namely *GhKAR*, *GhHAD*, and *GhENR*, play important roles in oil accumulation in cotton seeds. In the present study, these three genes were cloned and characterized. The expression patterns of *GhKAR*, *GhHAD*, and *GhENR* in the high seed oil content cultivars 10H1014 and 10H1041 differed somewhat compared with those of 10H1007 and 2074B with low seed oil content at different stages of seed development. *GhKAR* showed all three cultivars showed higher transcript levels than that of 2074B at 10-, 40-, and 45-days post anthesis (DPA). The expression pattern of *GhHAD* showed a lower transcript level than that of 2074B at both 10 and 30 DPA but a higher transcript level than that of 2074B at 40 DPA. *GhENR* showed a lower transcript level than that of 2074B at both 15 and 30 DPA. The highest transcript levels of *GhKAR* and *GhENR* were detected at 15 DPA in 10H1007, 10H1014, and 10H1041 compared with 2074B. From 5 to 45 DPA cotton seed, the oil content accumulated continuously in the developing seed. Oil accumulation reached a peak between 40 DPA and 45 DPA and slightly decreased in mature seed. In addition, *GhKAR* and *GhENR* showed different expression patterns in fiber and ovule development processes, in which they showed high expression levels at 20 DPA during the fiber elongation stage, but their expression level peaked at 15 DPA during ovule development processes. These two genes showed the lowest expression levels at the late seed maturation stage, while *GhHAD* showed a peak of 10 DPA in fiber development. Compared to 2074B, the oil contents of *GhKAR* and *GhENR* overexpression lines increased 1.05~1.08 folds. These results indicated that *GhHAD*, *GhENR*, and *GhKAR* were involved in both seed oil synthesis and fiber elongation with dual biological functions in cotton.

## 1. Introduction

Cotton (*Gossypium hirsutum* L.) is an important commercial crop that is grown worldwide as a source of fiber and edible oil [1,2,3,4]. Cotton fiber serves as an important raw material in the global textile industry and is a better alternative to synthetic fiber [5,6,7,8,9]. Cotton seed contains 28–40% oil, which could be used as edible oil and raw material for chemical production as well as a feedstock for biodiesel production [5,10]. Refined as edible oil, cottonseed oil is typically composed of about 26% palmitic acid (C16: 0), 15% oleic acid (C18: 1), and 58% linoleic acid (C18: 2) [11], with less total serum cholesterol but more effectively usage compared with corn (*Zea mays*) oil [12], and provides several kinds of benefits and essential fatty acids. Therefore, cottonseed oil is a valued raw material in the food industry because it contains a high quantity of saturated palmitic acid and lacks unstable linolenic acids, imparting good stability and flavor properties [13].

Annually, billions of barrels of fossil oil are consumed worldwide, so cottonseed oil could also serve as a potential raw material for the energy industry as environmentally friendly biodiesel [14,15]. In view of this, dissecting the genetic basis of cottonseed oil formation and increasing its content should be paid more attention. Thus, developing cotton cultivars with high oil content with desired yield and fiber quality traits reserved are the further direction of breeding in cotton [16]. Researchers have found that overexpression of the cotton gene *GhDGAT1* with a seed-specific promoter in cotton seeds increased oil content to 13.9% in different transgenic lines [17]. Zhu et al. 2021 performed a comparative transcriptome analysis to identify the key genes for oil accumulation in cultivated tetraploid cotton [18]. They found three key transcription factors, WRI1, NF-YB6, and DPBF2, played a vital role in regulating seed oil in two tetraploid cotton. *SAD6* and *FATA* genes were more important for oil biosynthesis in *G. barbadense* than that in *G. hirsutum*. In addition, *GbSWEET* and *GbACBP6* could significantly increase the oil content in *G. barbadense* cotton [18]. Another study showed that the transcription factor GhHSL1 was involved in the regulation of cottonseed oil content based on the methods of quantitative trait loci (QTLs) and transcriptome analysis [19]. Bioinformatic and phylogenetic analyses also revealed that cotton stearoyl-acyl carrier protein desaturase (SAD) genes were involved in cottonseed oil biogenesis [20]. By comparative transcriptome analysis between two germplasms with the high-oil and low-oil contents of cottonseeds, a recent report found that *GhCYSD1* was identified as an important key player in oil biosynthesis. The overexpression of *GhCYSD1* in yeast resulted in increased oil content and altered fatty acid composition [21]. All these previously reported candidate genes might be used to increase oil content in cottonseed without affecting the yield and quality of cotton fiber.

However, in addition to the oil biosynthesis genes reported above, Type II fatty acid synthase (FAS) also catalyzes the synthesis of straight-chain fatty acids with 16 or 18 carbon atoms in cotton [22,23]. Cotton FASII consists of six kinds of enzymes: among them, β-ketone fatty acyl-ACP reductase (KAR), β-hydroxy fatty acyl-ACP dehydratase (HAD), and enoyl-ACP reductase (ENR) catalyzed the carbon-chain reduction, dehydration, and reduction reactions [24,25]. In *Eustigmatos* cf. *polyphem* (an oleaginous microalga), transcriptome data indicated that *KAR*, *HAD*, and *ENR* were key genes in fatty acid biosynthesis and metabolism [26]. Compared to date palm, the total transcript levels of *KAR*, *HAD*, and *ENR* at five stages of mesocarp development were significantly higher in the oil palm by 44, 34, and 17-fold, respectively, based on the transcriptome sequencing method, indicating that these three genes were involved in the regulation of oil content in the mesocarp tissue of palms [27]. Another report observed that *KAR*, *HAD*, and *ENR* showed consistent transcription level trends at four stages of seed development in four oilseed plants [28]. In peanuts, analysis of the transcript levels of *AhKAR*, *AhHAD*, and *AhENR* genes at different stages of ovule development showed that the expression pattern of *AhHAD* differed from those of *AhKAR* and *AhENR* [29].

In cotton, the full-length cDNAs of *GhKAR*, *GhHAD*, and *GhENR* have been cloned, and each encodes a 283, 221, and 394 amino acid protein, respectively. Bioinformatic analysis of *GhKAR*, *GhHAD*, and *GhENR* indicated that the three genes served important functions in oil accumulation and were involved in the response to physiological stress [30]. To deeply provide new insights into understanding the mechanism of *GhKAR*, *GhHAD*, and *GhENR* in regulating the fatty acid biosynthesis network, in this study, we examined the expression patterns of *GhKAR*, *GhHAD*, and *GhENR* in cotton during fiber and seed development. We found that the expression levels of *GhKAR*, *GhHAD*, and *GhENR* in high seed oil content cultivars differed from the low seed oil content cultivars at different stages of seed development. Thereinto, *GhKAR* and *GhENR* presented different expression patterns during fiber and ovule development processes. Overexpression *of GhKAR* and *GhENR* slightly increased the oil contents in cottonseeds. These results not only bring new perspectives for understanding the mechanism of seed oil accumulation and fiber quality in cotton but also provide three new genes for the potential increase in oil content in cottonseeds by genetic engineering methods.

## 2. Results

### 2.1. Identification of Overexpressing GhKAR, GhHAD and GhENR Genes Plants

**Figure 1:**
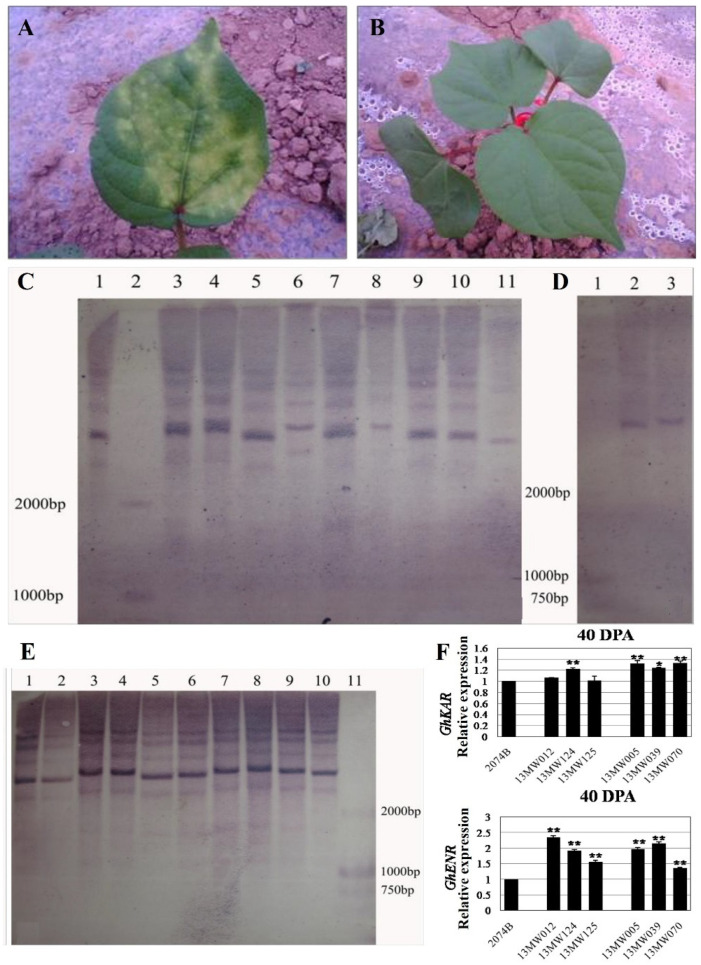
Identification of overexpressing *GhKAR*, *GhHAD* and *GhENR* genes plants. (**A**) transgenic negative plants identified by kanamycin. (**B**) transgenic-positive plants identified by kanamycin. (**C**) Southern blotting detection of transgenic *GhENR* plants. One, 3–11 were the results of *GhENR* and 2 was Marker D2000; (**D**) Southern blotting detection of transgenic *GhHAD* plants. One: Marker D2000; 2 and 3: Southern blotting results of *GhHAD* plants. (**E**) Southern blotting detection of transgenic *GhKAR* plants. One–Ten: Southern blotting results; 11: Marker D2000. (**F**) Expression levels of *GhKAR* and *GhENR* at 40DPA ovules in six T_3_ transgenic lines that overexpression *GhKAR* (13MW012, 13MW124, and 13MW125) or *GhENR* (13MW005, 13MW039, and 13MW070) compared with the receptor cultivar 2074B (means of triplicates ± SD, * *p* < 0.05, ** *p* < 0.01, Student’s *t*-test).

We primarily used a 5 g/L kanamycin solution to identify the transgenic cotton plants. After 3–5 days of application on leaves, those with yellow spots on the leaves were transgenic-negative plants (Figure 1A), while those without yellow spots were transgenic-positive candidate plants (Figure 1B). Then, the genomic DNA of transgenic positive plant leaves was extracted and digested by BamHI-HF endonuclease. The labeled probe was the fragment of kanamycin resistance encoding gene Kan. The copy number of transgenic positive plants was identified by the Roche Southern blot kit, and it was found that the copy number of the labeled probe in transgenic plants was between 1–4, as shown in Figure 1C–E. The results showed that *GhKAR* and *GhENR* overexpressed lines contained three and four copy transgene insertions (Figure 1C,E), while the copy number of overexpressed *GhHAD* plants was single (Figure 1D). The qRT-PCR analysis of 40DPA ovules revealed that the expression levels of six T_3_ transgenic lines that overexpression *GhKAR* (13MW012, 13MW124, and 13MW125) or *GhENR* (13MW005, 13MW039, and 13MW070) were all higher than the receptor cultivar 2074B (Figure 1F).

### 2.2. Phenotypic Traits of the Plant Materials

Based on the seed oil content and gene copy number, four wild-type cotton cultivars (2074B, 10H1007, 10H1014, and 10H1041) and six T_3_ transgenic lines that overexpression *GhKAR* (13MW012, 13MW124, and 13MW125) or *GhENR* (13MW005, 13MW039, and 13MW070), each with single gene copy and high seed oil content, were selected to study the functions of *GhKAR*, and *GhENR* in cotton oil metabolism (Table 1). The results showed that two wild-type cotton cultivars (10H1014 and 10H1041) contained high oil contents up to 30.73% and 35.97%, respectively. However, another two wild types (10H1007 and 2074B) contained oil contents low to 26.09% and 27.32%, respectively. In this study, 2074B was the background material for the transgene receptor. Compared to 2074B, the oil contents of three *GhKAR* overexpression lines slightly increased by 1.05~1.07 folds, and *GhENR* overexpression lines slightly increased by 1.06~1.08 folds with no significant differences.

The other important agronomic and economic traits of four wild-type cotton cultivars were also recorded. Among the four cultivars, 10H1041 was distinct, with a plant height of 86.8 ± 4.73 cm and the height of the first branch at 18.3 ± 2.21 cm, which were significantly lower than the others (Table 2). As for the six transgenic lines, the plant height, height of the first branch, branch number, and lint percentage were significantly higher, whereas the seed indices were significantly lower than those of the control line 2074B (Table 3). Among them, 13MW125 was a favorable line in which the plant height, seed oil content, and lint percentage were significantly higher compared with those of 2074B; however, its fiber quality was not significantly different (Table 3).

### 2.3. Morphological Changes in Immature Ovules

Similar morphologically immature ovules at different stages were indistinguishable as seed maturity differed among bolls collected from different parts of the same plant. After the ovules were shelled and dried, the morphology of the ovules at different seed developmental stages differed notably. The sizes of dried ovules at different developmental stages between 10H1007 and 2074B were consistent (Figure S1A). Similarly, the sizes of dried ovules at different developmental stages also showed no significant difference between the *GhKAR*-overexpression line (13MW125), *GhENR*-overexpression line (13MW039), and 2074B (Figure S1B,C).

### 2.4. Moisture Content, Grain Weight, and Oil Content of Four Wild-Type Cotton Cultivars and Six T_3_ Transgenic Lines during Seed Development

Seed samples from the four wild-type cotton cultivars were collected at 20, 25, 30, 35, 40, and 45 DPA and mature stage. The seed moisture content at each developmental stage was determined. The changes in seed moisture content at the different seed developmental stages were similar among the four wild-type cotton cultivars (Table 4). The moisture content of ovules ranged from 81% to 87% at 20 and 25 DPA, 71–76% at 30 DPA, 61–65% at 35 DPA, about 55–59% at 40 DPA and 46–50% at 45 DPA. However, at maturity, the moisture content of ovules had decreased to 5–6%. When the cotton bolls developed after 45 DPA, the seeds were already mature. However, between 45 DPA and the maturity stage, the moisture content decreased by almost 50%, suggesting that seed ripening occurred.

Moreover, the grain weight in four wild-type cotton cultivars increased by about 1 g every 5 d, but there existed no significant correlations between the grain weight and oil content in each line (Table 4). As also shown in Table 4, there were similar oil content accumulation trends among the four wild-type cultivars with different accumulation rates. Oil accumulation rapidly increased in ovules during the period of 20–30 DPA and peaked at 40 and 45 DPA, then decreased slightly at the mature stage. Among the four cultivars, 10H1041 contained the highest oil content, with oil content declining less at the maturity stage than that in the other three lines.

At the 20 DPA and 25 DPA, most grain weights of the six T_3_ transgenic lines were significantly higher than that of 2074B (Table 4). However, at the late developmental stages (30–45 DPA), most grain weights of the six T_3_ transgenic lines were significantly lower than that of 2074B, which were mainly caused by the decreased moisture content at the late stages (Table 4).

The oil contents of *GhKAR, GhHAD,* and *GhENR* overexpression transgenic cotton lines presented similar patterns (Table 4). The oil content of 13MW125 was the highest among the six transgenic lines at the mature stage and could be used as an important germplasm resource material. The net growth rate of oil content in most *GhKAR* and *GhENR* overexpression transgenic lines was higher than their receptor material (2074B) at 30–35 DPA (Table 5), indicating that *GhKAR* and *GhENR* may have important functions in oil accumulation at 30–35 DPA.

### 2.5. Expression Patterns of GhKAR, GhHAD, and GhENR in 2074B

**Figure 2:**
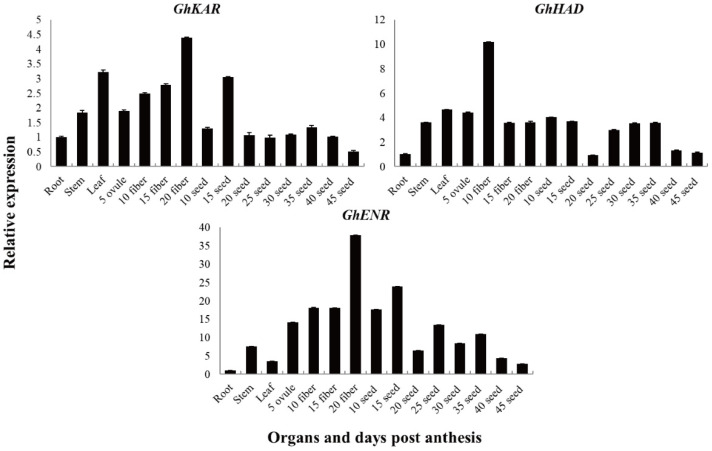
Transcription patterns of *GhKAR*, *GhHAD*, and *GhENR* in vegetative organs, and during fiber and seed development in upland cotton 2074B. Numerals in fiber and ovule development stages indicate the number of days post-anthesis; the values and error bars the mean +/− SE of three biological replicates.

The expression patterns of *GhKAR*, *GhHAD*, and *GhENR* genes in different tissues were analyzed using quantitative real-time RT-PCR (qRT-PCR). To investigate the expression patterns of these three genes, which are associated with fatty acid synthesis and metabolism, in 2074B (Figure 2), total RNAs isolated from the roots, stems, leaves, fibers (5, 10, 15, and 20 DPA), and seeds (10, 15, 20, 25, 30, 35, 40, and 45 DPA), were used as templates, respectively. The housekeeping gene *GhUBQ7* was used as an internal comparison gene.

The genes *GhKAR*, *GhHAD*, and *GhENR* were constitutively expressed. The transcript levels of *GhKAR* and *GhHAD* were higher in the leaves than in the stems and roots, whereas the transcript levels of *GhENR* were significantly higher in the stems than in the leaves and roots (Figure 2). The transcript levels of *GhKAR* and *GhENR* were high in the fiber (especially in 20 DPA fiber tissue) and 15 DPA seeds. We, therefore, speculated that the expression of *GhKAR* and *GhENR* has important functions in seed oil synthesis and fiber elongation. The transcript level of *GhHAD* was high in fibers and seeds and especially high in 10 DPA fibers. *GhHAD* may have a similar function as *GhKAR* and *GhENR* but showed a different expression pattern.

### 2.6. Expression Patterns of GhKAR, GhHAD, and GhENR in Four Cotton Cultivars

#### 2.6.1. Expression Patterns in Fibers

**Figure 3:**
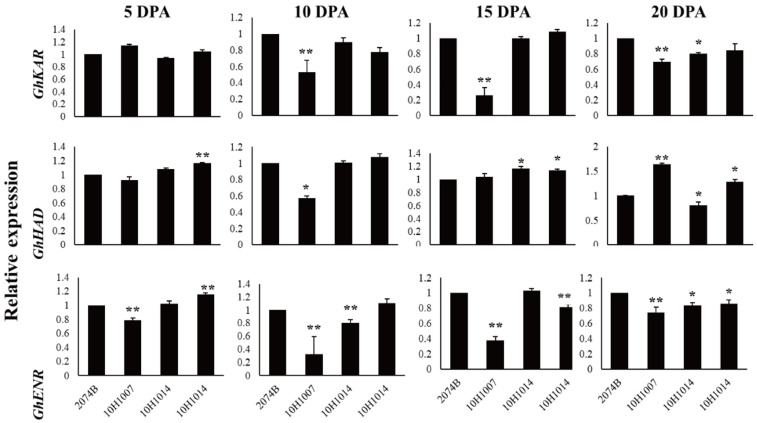
Expression levels of *GhKAR*, *GhHAD*, and *GhENR* at different stages of fiber development in four non-transgenic cotton lines showing different seed oil contents. Values presented are the mean ± SE of three biological replicates. * and ** indicate significant differences in test of statistics (*p* < 0.05 and *p* < 0.01, respectively) compared with the 2074B value set at 1. Oil contents of the four lines were: 2074B, 26.41%; 10H1007, 28.09%; 10H1014, 30.88%; 10H1041, 35.29%. DPA, days post-anthesis.

The expression patterns of *GhKAR*, *GhHAD*, and *GhENR* in fibers (5, 10, 15, and 20 DPA) were analyzed using qRT-PCR with the housekeeping gene *GhUBQ7* as the internal comparison gene. Among the four materials, the expression patterns of the three genes varied during fiber development. The expression pattern of *GhKAR* and *GhENR* both showed an increasing trend that peaked at 20 DPA. However, the expression pattern of *GhHAD* differed with peaking at 10 DPA (Figure 3).

#### 2.6.2. Expression Patterns in Ovules

To investigate the gene expression patterns in relation to ovule development, the expression patterns of *GhKAR*, *GhHAD*, and *GhENR* genes in ovules (5, 10, 15, 20, 25, 30, 35, 40, and 45 DPA) were also analyzed using qRT-PCR with the housekeeping gene *GhUBQ7* as an internal comparison gene.

**Figure 4:**
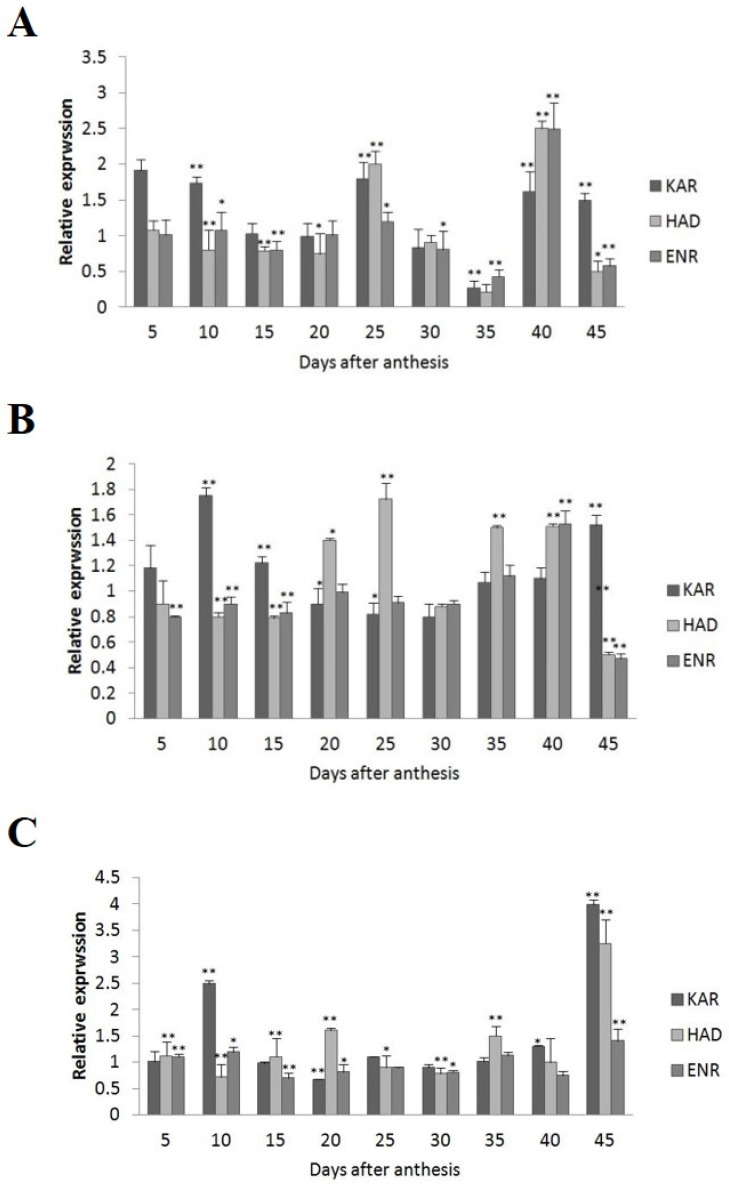
Expression levels of *GhKAR*, *GhHAD*, and *GhENR* at different stages of ovular development in three cotton cultivars showing different seed oil contents. (**A**) 10H1014; (**B**) 10H1007; (**C**) 10H1041. Values presented are the mean ± SE of three biological replicates. * and ** indicate significant differences in test of statistics (*p* < 0.05 and *p* < 0.01, respectively) compared with the 2074B value set at 1. Seed oil contents of the four lines were: 2074B, 26.41%; 10H1007, 28.09%; 10H1014, 30.88%; and 10H1041, 35.29%. DPA, days post-anthesis.

Among the four wild-type cultivars, seed oil contents were 26.41% in 2074B, 28.09% in 10H1007, 30.88% in 10H1014, and 35.29% in 10H1041. Meanwhile, the expression patterns of *GhKAR*, *GhHAD*, and *GhENR* genes were analyzed in developing ovules of the three wild-type cultivars compared to 2074B (Figure 4). Compared to the 2074B, *GhKAR* showed high expression levels at 5, 10, 25, 40, and 45 DPA, while the transcript levels of *GhHAD* and *GhENR* were higher at 25 and 40 DPA in 10H1014 (Figure 4A). As for 10H1007, the transcript level of *GhKAR* was almost two-fold 2074B at 10, 15, and 45 DPA; *GhHAD* showed high expression levels at 20, 25, 35, and 40 DPA, while *GhENR* only increased its transcript level at 40 DPA (Figure 4B). For 10H1041, *GhKAR* was expressed highly at 10, 40, and 45 DPA, *GhHAD* elevated its expressions at 20, 35, and 45 DPA, and *GhENR* increased only at 45 DPA compared with 2074B (Figure 4C). The above results showed that *GhKAR*, *GhHAD*, and *GhENR* genes almost presented higher expression levels at 10H1007, 10H1014, and 10H1041 cultivars with higher seed oil contents than 2074B. However, at this stage, the cultivars that showed high gene transcript levels were not necessarily consistent with the cultivars that showed low oil contents. Interestingly, at the late stage of oil accumulation (40–45 DPA), cultivars with high oil contents exhibited high gene transcript levels. We, therefore, hypothesized that the high transcript levels of *GhKAR*, *GhHAD*, and *GhENR* at the late oil accumulation stage may affect oil accumulation.

## 3. Discussion

### 3.1. Genes Related to the Oil Biosynthesis and Accumulation in Cottonseed

Cotton is a leading natural resource for fiber and oil. A previous study investigated the genetic architecture of seed nutrients in upland cotton by a genome-wide association study (GWAS) method with a panel of 196 germplasm resources. They identified three candidate genes, *Gh_D12G1161*, *Gh_D12G1162,* and *Gh_D12G1165,* that were most likely involved in the formation of cottonseed protein and fatty acid compositions [31]. Another study used a genetic map with 388 molecular markers to identify QTL for oil content in cottonseed. Two candidate genes, *Gh_A03G0701* and *Gh_A03G0699*, were screened at the overlapped qOil-3 region. These two genes encoded the 3-ketoacyl-CoA enzyme, which was closely related to the synthesis of oil in cottonseeds [32]. QTL-mapping and regulatory network analyses also suggested that *Ghr-miR2949b*, *Ghr-miR2949c*, and *GhHSL1* were closely involved in the cottonseed oil content [19]. In addition, 83 representative upland cotton accessions grown in multiple environments were used to identify the quantitative trait loci (QTLs) underpinning cottonseed oil content and fatty acid components. Finally, three genes were screened as the candidate genes, and the ectopic expression of *Gh_D01G2016* in yeast led to the sharp reduction in oil content, which suggested that the function of this gene could be verified by gene editing technology in oil biosynthesis of cottonseeds [33]. These results have widened our understanding of the genetic mechanism on cottonseed oil formation and provided important molecular tools to develop new cultivars with high fatty acid contents in cotton breeding by marker-assisted selection method.

With the advent of numerous high-quality genome sequences and omics technology platforms from *Gossypium* species, key genes involved in oil biosynthesis in cottonseeds have been identified. For instance, transcriptome data showed that *WRI1*, *NF-YB6*, and *GhPDAT* genes play important roles in the oil accumulation in fatty acid (FA) synthesis, FA desaturation, and triacylglycerol (TAG) biosynthesis [34]. Other early reports found that *GhWRI1* was involved in oil metabolism. *GhWRI1* showed high expressions in high oil content material, and overexpression of *GhWRI1* increased seed oil content in transgenic *A. thaliana*, suggesting its crucial role in seed oil accumulation [35]. In addition, phosphoenolpyruvate carboxylase genes were also the potential targets for creating high cottonseed oil materials by RNAi strategy in cotton. For example, the cottonseed oil content in *GhPEPC1* RNAi lines showed a significant increase without other phenotypic changes, and decreasing the *GhPEPC1* expression led to the increased expression of triacylglycerol biosynthesis-related genes, which might contribute to the oil biosynthesis in cottonseeds [36]. Silencing the *GhPEPC2* gene using RNAi could also increase oil accumulation in cotton seeds [37]. All these candidate genes could serve as the foundation for elucidating the molecular mechanisms of oil content formation and the genetic breeding for higher cottonseed oil content.

Genome-wide level analyses indicated that the omega-3 FAD gene family in cotton was characterized to be differentially expressed in seeds [38]. Meanwhile, the expression levels of *GhCPS1* and *GhCPS2* genes closely correlated with the total cyclopropenoid fatty acids (CFA) content in cottonseeds, and both of them can be considered potential targets for gene silencing to reduce undesirable seed CPE accumulation in cotton [39]. Another study showed that LPAAT genes were co-localized with quantitative trait loci (QTL) region for cottonseed oil. Overexpression of one LPAAT gene, *Gh13LPAAT5*, significantly increased the production of total TAG and other fatty acids [40]. Additionally, SNP variations from *GhCIPK*s genes were significantly associated with oil content in cotton, and overexpression of the *GhCIPK6* gene reduced the oil content but increased C18:1 content in transgenic cotton [41]. These studies provided incentives for further underlying molecular mechanisms of oil accumulation in cottonseed oil but also brought new genes to increase cottonseed oil content through biotechnology.

### 3.2. The Application of Fatty Acid Synthase in Improving Seed Oil Content

Overexpression of *Spinacia oleracea KASIII* (*SoKASIII*) in tobacco leaves under the control of the 35S CaMV promoter and of *Jatropha Curcas KASIII* (*ChKASIII*) in *A. thaliana* and oilseed rape seeds under the control of the napin promoter, indicates that the 16:0 fatty acid contents of both transgenic plants were significantly increased [42]. In recent years, efforts have been made to manipulate key fatty acid synthetic genes in various species using transgenic technology [43,44,45,46,47]. Transformations using transgenes encoding key enzymes or enzyme subunits have resulted in the alteration in lipid levels to varying degrees [37,48], and in some cases, the oil content was reduced [49]. Four key enzymes were involved in the fatty acid carbon-chain extension process, namely KAS, KAR, HAD, and ENR. KAS can be divided into three categories (KASI, KASII, and KASIII) based on its catalytic substrates. KASIII serves an important function in the synthesis of C16:0 fatty acids [49]. KASII affects the C16:0 fatty acid content [50]. The mutation or deletion of KAS will affect not only the fatty acid synthesis but also the seed development [50,51]. Studies on KAR, HAD, and ENR were relatively limited. Transcriptional research works have shown that KAR, HAD, and ENR were involved in seed oil and fatty acid synthesis [27]. The ENR deletion mutant of *A. thaliana* suffered seed abortion and decreased fatty acid content. The deletion mutant of ENR in *A. thaliana* will cause seed abortion and fatty acid content decrement [30]. The mechanisms by which KAR, HAD, and ENR affect seed oil synthesis remain unclear; therefore, further studies are needed.

### 3.3. GhKAR, GhHAD, and GhENR Were Key Enzymes in the Synthesis of Fatty Acids

Oil accumulation is part of the seed maturation process, a highly controlled developmental program that sets in ovule tissues once morphogenesis has been achieved. The maturation process was characterized by the accumulation of storage compounds, acquisition of desiccation tolerance, and entry into a dormancy period of variable length [52]. Gene expression programs associated with these processes were activated during the maturation phase and were switched off during the vegetative phases of plant development. Studies of developing seeds and/or embryos have established that the biosynthetic pathways for fatty acids and TAGs were regulated at the transcription level [53,54,55]. In the present study, four cotton cultivars with different seed oil contents showed consistent oil accumulation trends during seed development. The overall trend of oil accumulation was that at 20–30 DPA, the oil content rapidly increased and thereafter showed slow accumulation. At 40 DPA, the oil content peaked and then slightly decreased. The decrease in oil content at maturity may be attributable to β-oxidation and several other physiological and biochemical processes, suggesting that the decline in oil accumulation during seed maturation might be overcome using molecular breeding techniques to achieve the goal of developing lines with high seed oil contents.

*GhKAR*, *GhHAD*, and *GhENR* were crucial genes involved in fatty acid carbon-chain extension that have been cloned from immature ovules of upland cotton [27]. The present analysis of transcript levels of these genes in different tissues and organs of 2074B indicated that the genes were highly expressed during fiber development and showed higher transcription levels than in most developing seeds. Therefore, these genes were not only involved in plant seed oil synthesis but also in the cotton fiber elongation process. The transcript levels of *GhKAR* and *GhENR* were particularly high at 20 DPA during fiber development and at 15 DPA during seed development. However, the transcript level of *GhHAD* was high only at 10 DPA during fiber development, which indicated that *GhHAD*, together with *GhKAR* and *GhENR*, showed different expression patterns during seed and fiber development. Analysis of the fatty acid compositions of the homozygous transgenic T_3_ and T_4_ generations of *A. thaliana* seed showed that overexpression of *GhKAR*, *GhHAD*, or *GhENR* could increase the total content of fatty acids in the seed. The total fatty acid content of transgenic *GhKAR*, *GhHAD*, and *GhENR* lines were 6.59%, 7.76%, and 3.86% higher than that of wild-type *A. thaliana*, respectively. Thus, overexpression of *GhKAR*, *GhHAD*, or *GhENR* may improve the total fatty acid content by increasing the concentration of every fatty acid because *GhKAR*, *GhHAD*, and *GhENR* are genes involved in fatty acid synthesis. Further studies were needed to verify this hypothesis and the molecular functions of the three genes.

## 4. Materials and Methods

### 4.1. Plant Materials and Growth Conditions

Four cotton cultivars (2074B, 10H1004, 10H1007, and 10H1041), and T_3_ transgenic cotton lines that overexpression *GhKAR* (13MW012, 13MW124, and 13MW125) and *GhENR* (13MW005, 13MW039, and 13MW070), were used in our experiments.

Plants were grown on soil in a greenhouse (16 h light/8 h dark) at 28 °C. After three weeks, the roots, stems, and leaves of seedlings at the three-leaf stage were harvested, frozen in liquid nitrogen, and stored at −80 °C for DNA and total RNA extraction. Seeds were grown in the field in Hejian, from which samples of fibers (5, 10, 15, and 20 DPA), and ovules (5, 10, 15, 20, 25, 30, 35, 40, and 45 DPA) of the different materials were collected.

### 4.2. Field Experiments and Agronomic Trait Investigation

For field experiments, non-transgenic and transgenic plants were sown directly on April 27th at Hejian, Hebei Province (38°43′ N, 116°09′ E). The field planting followed a randomized complete block design with three replications. Two-row plots with 80 cm and 50 cm row spacing were used. The length of each plot was 4m. Field management followed conventional standard field practices. Data were collected from at least 10 plants in each line. Self-pollinated bells were harvested from each primary transgenic (T_3_) plant and analyzed for quality character of fibers and seed oil content. The cottonseed oil content was determined using the Soxhlet extraction method and near-infrared spectroscopy [30]. All results were statistically analyzed by three times repeats. LSD test and difference significance test of statistics method were adopted in final result analysis.

### 4.3. RNA Isolation and cDNA Synthesis

Total RNA was extracted using a modified CTAB-SDS method [56]. RNA samples were treated with DNase I (Ambion, Austin, TX, USA) in accordance with the manufacturer’s instructions to remove genomic DNA contaminants. Total RNA samples (1 µg per reaction) were reverse transcribed into cDNA by avian myeloblastosis virus (AMV) reverse transcriptase. The cDNAs were used as the template in subsequent qPCR reactions.

### 4.4. Quantitative Real-Time RT-PCR

Total RNA was extracted from young leaves, roots, stems, and developing fibers (5, 10, 15, and 20 DPA) and ovules (5, 10, 15, 20, 25, 30, 35, 40, and 45 DPA) as indicated above. Gene-specific primers were designed to amplify PCR products of ~200 bp in length (see Supplementary Table S1). The relative level of gene expression was estimated using the 2^−△△CT^ method [57]. The analyses were performed with three biological replicates using samples from different plants. The SYBR^®^ Premix Ex Taq™ II (Tli RNaseH Plus) (TaKaRa, Biotechnology (Dalian, China) Co., Ltd.) was used for RT-PCR.

The expression patterns of *GhKAR*, *GhHAD*, and *GhENR* in different tissues were studied using qRT-PCR. To investigate the expression patterns of the three genes in relation to fatty acid synthesis and metabolism in 2074B, total RNAs isolated from the root, stem, young leaves, and ovule, as well as from fibers (5, 10, 15, and 20 DPA), and ovules (5, 10, 15, 20, 25, 30, 35, 40, and 45 DPA), of 2074B were used as templates. In addition, *G. hirsutum UBQ7* gene used to normalize served as an endogenous reference. Data are presented as the means (±SD) of three independent experiments.

### 4.5. Cotton Transformation

The coding sequences of *GhKAR*, *GhHAD*, and *GhENR* genes were amplified with the *Long and Accurate* polymerase (*TaKaRa*, Tokyo, Japan) from cDNA library and then inserted into the pCAMBIA 2301 vector. Cotton transformation was conducted using the pollen tube pathway method with cv. Sumian 20 as a receptor [58,59,60]. In brief, the flowers pollinated for 36–48 h were selected, and 0.1~0.2 μg plasmid DNA dissolved in double distilled water was injected into each ovule. The bolls injected with plasmid DNA were tagged. The T_1_ seeds of the transgenic plants were harvested and planted in the field, and then the transformants were primarily discriminated by checking the resistance to kanamycin (2 g/dm^3^) by spraying in the field during cotyledon and three leaves stages. The T_1_ plants showing kanamycin resistance were further selected for PCR and Southern blot analyses.

### 4.6. Identification of the Phenotypic Traits

Plant height was recorded by measuring the main stem height of individuals [61]. Fiber quality traits, including the fiber length (mm), fiber uniformity ratio (%), fiber strength (cN/tex), fiber elongation, and micronaire, were measured with an HVI 900 instrument (USTER HVISPECTRUM, SPINLAB, USA) at the Cotton Fiber Quality Inspection and Test Center of Ministry of Agriculture (Anyang, China) [62]. Boll samples were ginned for seed index, and one hundred cottonseeds were randomly selected from each line and weighed as seed index (SI, g) [14]. Cottonseeds delinted with concentrated sulphuric acid were used to measure oil content. The oil contents at the different stages of ovular development of the transgenic lines were measured using the Soxhlet extraction method [63]. The moisture content in cottonseed was analyzed by near-infrared reflectance spectroscopy (NIRS) technique [64]. Grain weight was recorded by weighing one hundred cotton ovules randomly.

### 4.7. Southern Blotting Analysis

Procedure for Southern blot was described briefly, as follows. Kan fragment was amplified by PCR (500 bp), and the concentration of recovered DNA was diluted to 60–70 ng/µL. Add 16 µL Kan fragment in a 200 µL centrifuge tube, bathe in boiling water for 10 min, quickly place on ice containing sodium chloride for 10 min, and centrifuge instantaneously. Adding 4 µL DIG-High-Prime, instantaneous centrifugation, and 37 °C water bath for 20 h; then, 2 µL 0.2 M EDTA (pH8.0) (or 10 min in 65 °C water bath) was added to terminate the reaction and stored at −20 °C. Probe denaturation: Add 5 µL labeled probe into 200 µL centrifuge tube, denaturate in water bath at 68 °C for 10 min after sealing with sealing film, and cool rapidly in ice water for 5 min (denaturation before use). The subsequent enzymatic digestion of cotton genomic DNA, treatment of digested products, electrophoresis, transmembrane and crosslinking, prehybridization and hybridization, rigor elution, detection reaction, and color reaction were finished according to the protocol of Roche Southern blot kit.

### 4.8. Statistical Analysis

The SPSS 13.0 statistical package (IBM Corporation, New York, NY, USA) was used for the analysis of variance and Student’s *t*-test. The normality test was checked by using the Shapiro-Wilk test to prove the data to satisfy the Gaussian distribution. The significance was tested using the least significant difference (LSD) at the 1% or 5% levels. Each sample included in the analysis was based on three biological replicates.

## 5. Conclusions

In this study, we observed that *GhKAR* and *GhENR* showed similar expression trends during fiber and seed development in three cultivars with different oil contents. The transcript levels of *GhKAR* and *GhENR* gradually increased and peaked at 20 DPA during fiber development. Considering that 10–20 DPA is a period of rapid fiber elongation, *GhKAR* and *GhENR* might have important functions at the late stage of rapid fiber elongation. However, the expression level of *GhHAD* peaked at 10 DPA, which suggested that *GhHAD* plays an important role in the early stage of rapid fiber elongation. Our results provided new insights into the fatty acid biosynthesis in cotton.

## Figures and Tables

**Table 1:** Oil content of the four wild-type and six T_3_ transgenic cotton lines used in the study.

Materials ID	Genotypes	Oil Contents/%
10H1041	wild-type	35.97 ± 0.88
10H1014	wild-type	30.73 ± 0.42
10H1007	wild-type	26.09 ± 0.58
2074B	wild-type	27.32 ± 0.96
13MW012	KAR OE line	28.56 ± 0.30
13MW124	KAR OE line	29.12 ± 0.36
13MW125	KAR OE line	28.87 ± 0.55
13MW005	ENR OE line	29.36 ± 0.54
13MW039	ENR OE line	29.61 ± 0.77
13MW070	ENR OE line	28.93 ± 0.46

Note: The oil content was determined by Soxhlet extraction method. OE: overexpression. Values were presented by means ± SD, *n* = 3.

**Table 2:** Phenotypic traits of the four non-transgenic cotton lines.

Traits	10H1041	10H1014	10H1007	2074B
Plant height (cm)	86.8 ± 4.73 ^c^	103.1 ± 16.88 ^ab^	109.9 ± 11.33 ^a^	92.9 ± 13.74 ^bc^
Height of first branch (cm)	18.3 ± 2.21 ^b^	30.5 ± 6.65 ^a^	29.7 ± 5.17 ^a^	28.9 ± 3.00 ^a^
Branch number	15.2 ± 1.14 ^a^	14.5 ± 1.72 ^ab^	15.9 ± 1.97 ^a^	12.9 ± 1.79 ^b^
Lint percent (%)	36.04 ± 1.55 ^b^	36.72 ± 0.82 ^b^	43.13 ± 1.51 ^a^	38.95 ± 1.22 ^ab^
Seed index (g)	11.70 ± 0.07 ^ab^	11.88 ± 0.02 ^a^	10.89 ± 0.61 ^b^	11.23 ± 0.34 ^ab^
Fiber length (mm)	29.63 ± 0.47 ^a^	29.76 ± 0.18 ^a^	29.40 ± 1.69 ^a^	30.63 ± 0.29 ^a^
Fiber uniformity ratio (%)	85.80 ± 0.28 ^a^	85.10 ± 1.98 ^a^	86.45 ± 1.06 ^a^	86.85 ± 0.21 ^a^
Fiber strength (cN/tex)	29.70 ± 0.57 ^b^	29.70 ± 0.14 ^b^	30.05 ± 1.06 ^ab^	31.45 ± 0.07 ^a^
Fiber elongation (%)	6.25 ± 0.07 ^a^	6.35 ± 0.07 ^a^	6.40 ± 0.14 ^a^	6.30 ± 0.00 ^a^
Micronaire	4.83 ± 0.31 ^a^	4.27 ± 0.15 ^a^	4.60 ± 0.28 ^a^	4.37 ± 0.19 ^a^

Note: Values followed by the same letters within the row are not significantly at the 0.05 significance level as indicated the LSD test. Values were presented by means ± SD, *n* = 30.

**Table 3:** Phenotypic traits of six transgenic cotton lines and non-transgenic cotton 2074B.

Traits	2074B	13MW012	13MW124	13MW125	13MW005	13MW039	13MW070
Plant height (cm)	92.9 ± 13.74	95.1 ± 8.20	108.9 ± 9.48 **	109.2 ± 10.03 **	99.6 ± 9.41	112.3 ± 4.81 **	103.4 ± 9.38
Height of first branch (cm)	28.9 ± 3.00	33.0 ± 6.88	36.3 ± 4.97 **	34.54 ± 9.14	31.2 ± 4.92	26.1 ± 3.28	27.7 ± 3.97
Branch number	12.9 ± 1.79	13.8 ± 2.35	13.8 ± 2.94	12.8 ± 4.34	13.4 ± 1.96	17.1 ± 0.88 **	15.1 ± 1.20 **
Lint percent (%)	38.95 ± 1.22	36.92 ± 2.57	39.78 ± 1.59	43.85 ± 2.06 **	39.18 ± 2.04	44.14 ± 1.71 **	37.29 ± 1.83
Seed index (g)	11.23 ± 0.34	10.73 ± 1.07	10.16 ± 0.80	10.01 ± 0.90	9.34 ± 0.80 **	9.64 ± 1.54	9.75 ± 0.73 *
Fiber length(mm)	30.63 ± 0.29	30.06 ± 0.48	29.63 ± 0.75	30.43 ± 0.80	30.97 ± 0.83	27.69 ± 0.93 *	30.81 ± 0.88
Fiber uniformity ratio (%)	86.85 ± 0.21	85.63 ± 0.86	85.57 ± 0.76	87.54 ± 0.83	85.85 ± 0.07 *	86.23 ± 0.46	84.94 ± 1.25
Fiber strength (cN/tex)	31.45 ± 0.07	33.27 ± 1.63	32.53 ± 0.67	31.14 ± 1.47	30.6- ± 0.71	29.13 ± 1.66	32.96 ± 0.68 *
Fiber elongation (%)	6.30 ± 0.00	6.13 ± 0.12	6.20 ± 0.17	6.26 ± 0.23	6.20 ± 0.00 **	6.48 ± 0.19	6.00 ± 0.07 **
Micronaire	4.37 ± 0.19	4.35 ± 0.62	4.34 ± 0.28	4.71 ± 0.28	4.03 ± 0.77	4.70 ± 0.41	3.96 ± 0.31

Note: * and ** indicate that the difference is significant at the 0.05 and 0.01 probability levels, respectively. Values were presented by means ± SD, *n* = 30.

**Table 4:** Phenotypic traits for the ovule of four wild-type cotton cultivars and six T_3_ transgenic lines.

Varieties Name	Plant Lines and Traits	20 DPA	25 DPA	30 DPA	35 DPA	40 DPA	45 DPA	Mature
10H1041	Moisture content (%)	86.15	85.05	76.90	65.01	55.05	46.96	5.12
	Grain weight (g)	1.52 ± 0.05 ^c^	1.95 ± 0.03 ^a^	2.91 ± 0.03 ^a^	4.37 ± 0.04 ^a^	5.66 ± 0.01 ^a^	7.06 ± 0.03 ^a^	
	Oil content (%)	1.37 ^d^	14.57 ^ab^	24.12 ^c^	32.53 ^a^	36.27 ^a^	35.29 ^a^	35.29 ^a^
10H1014	Moisture content (%)	83.12	84.80	71.17	65.42	56.76	50.74	4.57
	Grain weight (g)	1.74 ± 0.01 ^a^	1.81 ± 0.01 ^b^	2.90 ± 0.01 ^a^	4.15 ± 0.01 ^b^	4.29 ± 0.09 ^d^	5.70 ± 0.18 ^d^	
	Oil content (%)	6.25 ^a^	11.67 ^c^	29.31 ^a^	29.49 ^b^	33.55 ^b^	30.88 ^b^	30.88 ^b^
10H1007	Moisture content (%)	84.66	82.91	73.84	61.62	59.08	50.55	5.90
	Grain weight (g)	1.61 ± 0.01 ^b^	1.91 ± 0.01 ^a^	2.98 ± 0.02 ^a^	3.97 ± 0.02 ^c^	4.53 ± 0.04 ^c^	6.31 ± 0.01 ^c^	
	Oil content (%)	4.29 ^b^	14.03 ^b^	24.05 ^c^	28.31 ^c^	29.69 ^c^	24.72 ^c^	28.09 ^c^
2074B	Moisture content (%)	85.73	84.00	73.40	62.44	56.23	48.71	4.24
	Grain weight (g)	1.42 ± 0.02 ^d^	1.82 ± 0.02 ^b^	2.94 ± 0.11 ^a^	3.96 ± 0.05 ^c^	4.70 ± 0.05 ^b^	6.70 ± 0.05 ^b^	
	Oil content (%)	2.68 ^c^	15.06 ^a^	26.90 ^b^	29.52 ^b^	27.54 ^d^	29.86 ^b^	26.41 ^d^
13MW012 (*GhKAR* OE line)	Moisture content (%)	86.12	84.89	76.01	67.98	54.18	54.69	6.30
	Grain weight (g)	1.76 ± 0.01 **	1.94 ± 0.01 **	3.06 ± 0.01	3.98 ± 0.08	4.34 ± 0.01 **	6.44 ± 0.09 *	
	Oil content (%)	1.41 **	11.88 **	24.86 *	29.66	30.47 **	26.84 **	25.85
13MW124 (*GhKAR* OE line)	Moisture content (%)	87.02	85.44	75.10	68.67	55.83	52.92	5.97
	Grain weight (g)	1.42 ± 0.01	1.62 ± 0.01 **	2.90 ± 0.01	3.60 ± 0.05 **	4.90 ± 0.07 *	5.63 ± 0.03 **	
	Oil content (%)	1.42 **	12.69 *	27.50	28.94	30.44 *	26.37 *	27.18
13MW125 (*GhKAR* OE line)	Moisture content (%)	84.14	83.29	75.93	64.35	55.25	49.18	5.93
	Grain weight (g)	1.71 ± 0.01 **	2.04 ± 0.02 **	2.74 ± 0.01 *	3.85 ± 0.03 *	5.06 ± 0.01 **	5.54 ± 0.01 **	
	Oil content (%)	1.25 **	14.01 **	27.34	30.77 *	30.17 *	28.80	31.38 **
13MW005 (*GhENR* OE line)	Moisture content (%)	86.05	81.89	70.92	59.25	52.69	52.77	6.06
	grain weight (g)	1.51 ± 0.04 *	1.97 ± 0.04 **	2.98 ± 0.01	3.70 ± 0.03 **	4.64 ± 0.03	5.05 ± 0.02 **	
	Oil content (%)	3.73 **	16.47 **	25.43 *	30.45 *	29.41 *	27.20 **	27.77 *
13MW039 (*GhENR* OE line)	Moisture content (%)	86.25	83.74	76.67	64.52	52.03	48.82	4.48
	Grain weight	1.50 ± 0.03 *	1.71 ± 0.03 **	2.43 ± 0.02 **	3.60 ± 0.01 **	4.63 ± 0.01	5.05 ± 0.05 **	
	Oil content (%)	2.04 **	14.91	26.51	31.07 *	31.01 *	28.63 **	27.16
13MW070 (*GhENR* OE line)	Moisture content (%)	85.66	83.56	76.67	61.63	56.15	49.76	5.49
	Grain weight (g)	1.53 ± 0.03 **	1.86 ± 0.01	2.75 ± 0.03 *	3.82 ± 0.050 *	3.85 ± 0.02 **	5.31 ± 0.01 **	
	Oil content (%)	3.12 *	14.50	24.07 *	29.59	30.19 *	27.76 *	25.88

Note: Values followed by the same letter within a row are not significantly different at the 0.05 significance level as indicated by the LSD test. DPA, days post-anthesis. * and ** means data were significantly different at *p*-values of 0.05 and 0.01, respectively. Values were presented by means ± SD, *n* = 3.

**Table 5:** Net increment in oil content of ovules of T_3_ transgenic cotton lines at three stages of ovular development.

Material	Gene	Net Increment of 20DPA-25DPA (%)	Net Increment of 25DPA-30DPA (%)	Net Increment of 30DPA-35DPA (%)
2074B	Contrast	12.38 ± 0.05	11.84 ± 0.45	2.63 ± 0.18
13MW012	OEGhKAR	10.47 ± 0.01 **	12.99 ± 0.04	4.79 ± 0.27 *
13MW124		11.27 ± 0.62	14.81 ± 0.38 *	1.43 ± 0.19 *
13MW125		12.75 ± 0.11 *	13.33 ± 0.39	3.43 ± 0.03 *
13MW005	OEGhENR	12.74 ± 0.19	8.96 ± 0.09 *	5.02 ± 0.29 *
13MW039		12.87 ± 0.27	11.60 ± 0.75	4.57 ± 0.26 *
13MW070		11.38 ± 0.65	9.57 ± 0.86	5.53 ± 0.42 *

Note: * and ** indicate data are significantly different from contrast at *p*-values of 0.05 and 0.01, respectively. DPA, days post-anthesis. Values were presented by means ± SD, *n* = 3.

## Data Availability

Not applicable.

## Supplementary Materials

The following supporting information can be downloaded at: https://www.mdpi.com/article/10.3390/plants12193437/s1. Figure S1. Morphological changes in immature ovules of cotton at different developmental stages. (A) 10H1007 and 2074 (non-transgenic lines); (B) 13MW125 (*GhKAR*-overexpression line) and 2074B; (C) 13MW039 (*GhENR*-overexpression line) and 2074B. DPA, days post-anthesis. Figure S2. Phylogram of *GhKAR* protein sequences from different organisms; Figure S3. Phylogram of *Gh HAD* protein sequences from different organisms; Figure S4. Phylogram of *GhENR* protein sequences from different organisms; Table S1. Primers used in the experiment.

## Abbreviations

TAG: Triacylglycerol; FAS, Fatty acid synthase; β-ketone fatty acyl-ACP reductase (KAR); β-hydroxy fatty acyl-ACP dehydratase (HAD), and enoyl-ACP reductase (ENR); β-ketone fatty acyl-ACP synthase (KAS); Acetyl-CoA carboxylase (ACCase); acetyl-coenzyme A (CoA)-acyl carrier protein (ACP) transferase (ACAT); malonic acid single acyl-CoA-ACP transferase (MCAT); DPA, days post-anthesis; *G. hirsutum*, *Gossypium hirsutum*; *A. thaliana*, *Arabidopsis thaliana*; qRT-PCR, quantitative real-time polymerase chain reaction.
