# Supplementary material for: Genetic and Morpho-Physiological Differences among Transgenic and No-Transgenic Cotton Cultivars
Source: Plants (Basel). 2023 Sep 29;12(19):3437. doi: 10.3390/plants12193437 (PMC10574747; doi:10.3390/plants12193437)
Supplement: Supplementary file 1 [file plants-12-03437-s001.zip › Supplementary Table S1.pdf]

Supplementary Table S1 Primers used in the experiment

Pimer name	Primer sequence (5′→3′)	Explanation
GhK3F	CTGGCGTTAGAGGGAATGTGA	RT-PCR，Analysis of *GhKAR* expression
GhK3R	TCCAGGTAGTTGCCCACGATT	
GhH3F	GCCCATAATGCCTGGTGTTCT	RT-PCR，Analysis of *GhHAD* expression
GhH3R	GCTTTTGCAGCGTAATAAGTGTCA	
GhE3F	CGAAGCAGGAAGAAAGCACA	RT-PCR，Analysis of *GhENR* expression
GhE3R	CATAGCCGAAGCCAAAGGTG	
GhU7F	GAAGGCATTCCACCTGACCAAC	RT-PCR，housekeeping gene
GhU7R	CTTGACCTTCTTCTTCTTGTGCTTG	
