# Supplementary figures and images for: Genetic and Morpho-Physiological Differences among Transgenic and No-Transgenic Cotton Cultivars
Source: Plants (Basel). 2023 Sep 29;12(19):3437. doi: 10.3390/plants12193437 (PMC10574747; doi:10.3390/plants12193437)

**A**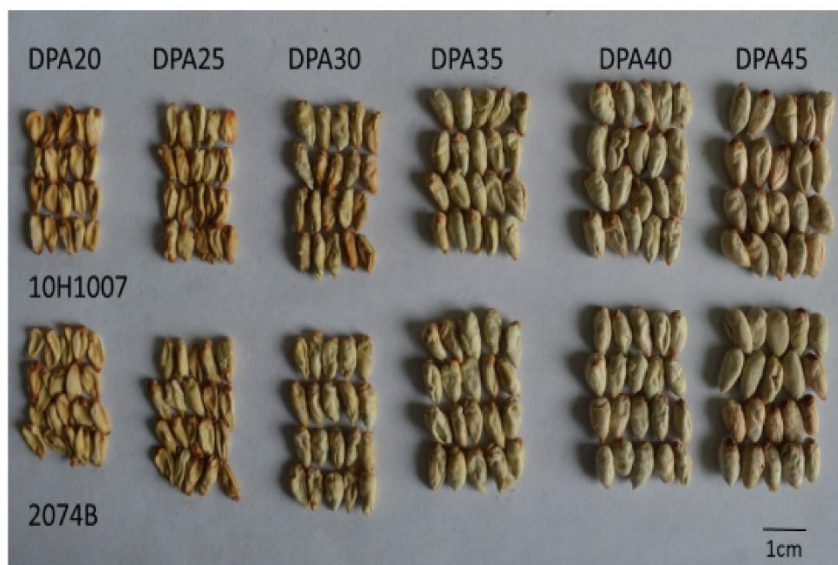**B**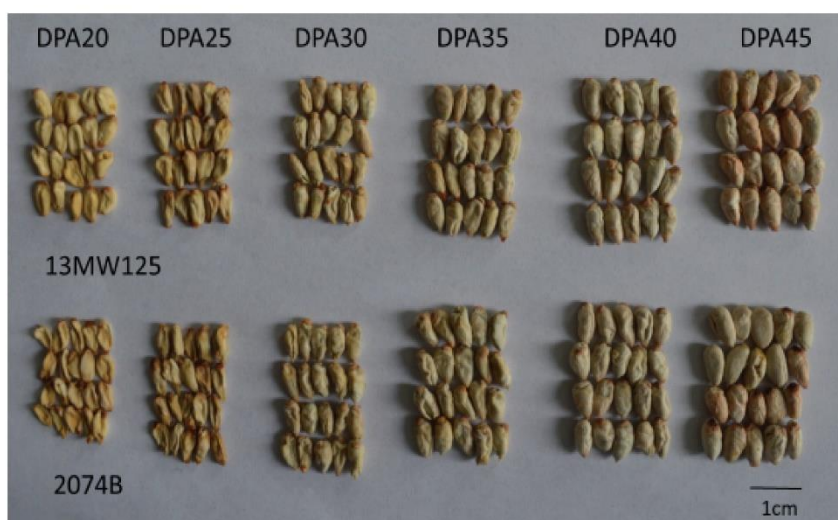**C**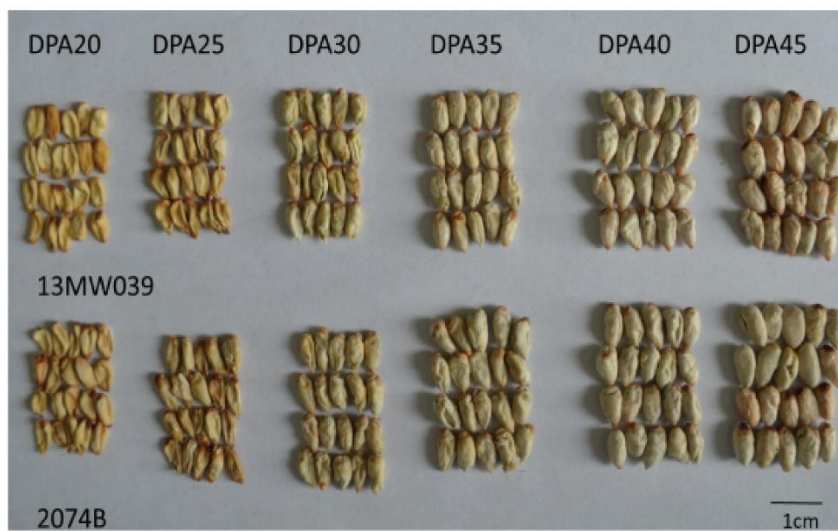

Supplement: Supplementary file 1 [file plants-12-03437-s001.zip › Figure S1.pdf]

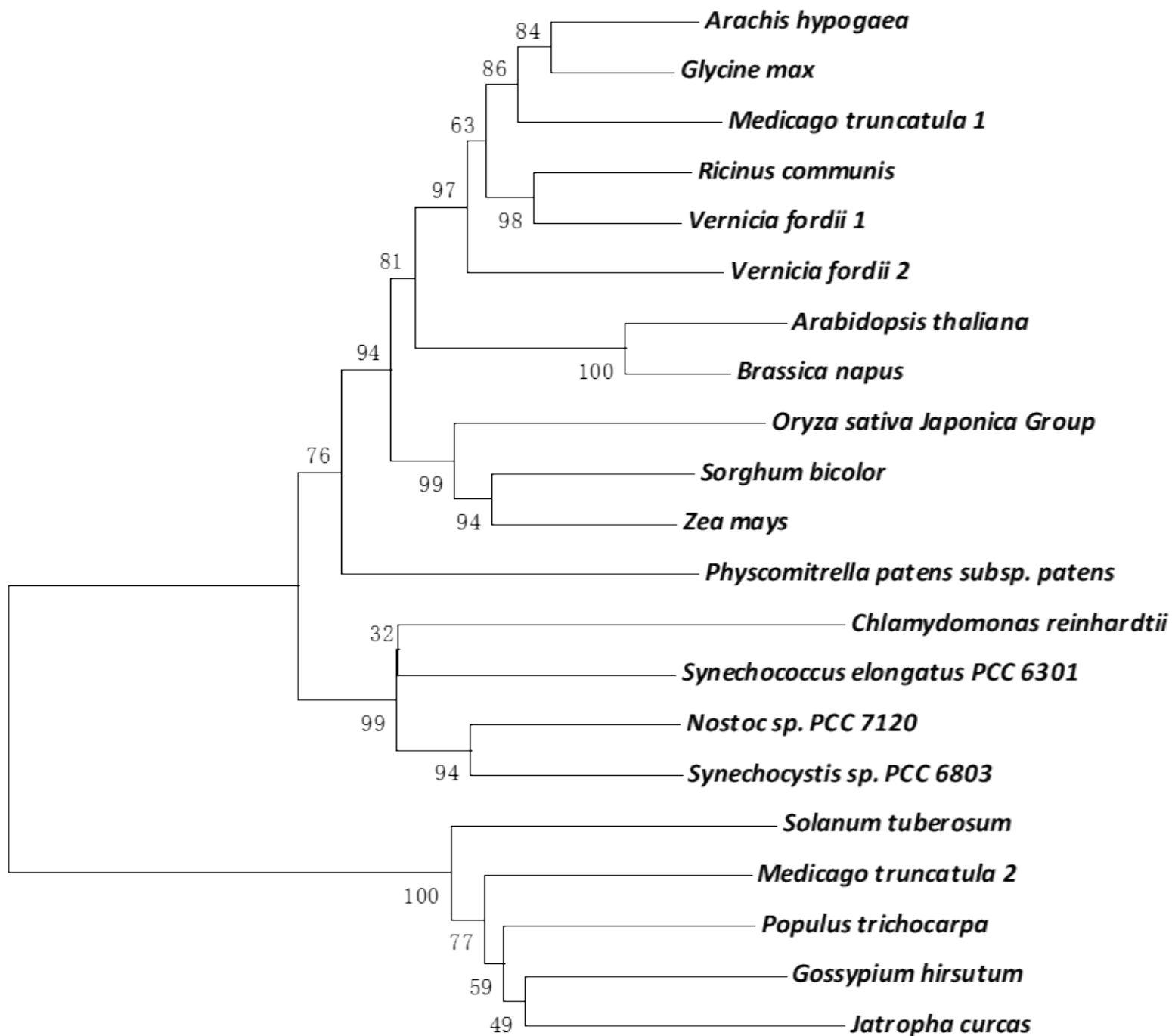

0.05

Supplement: Supplementary file 1 [file plants-12-03437-s001.zip › Figure S2.pdf]

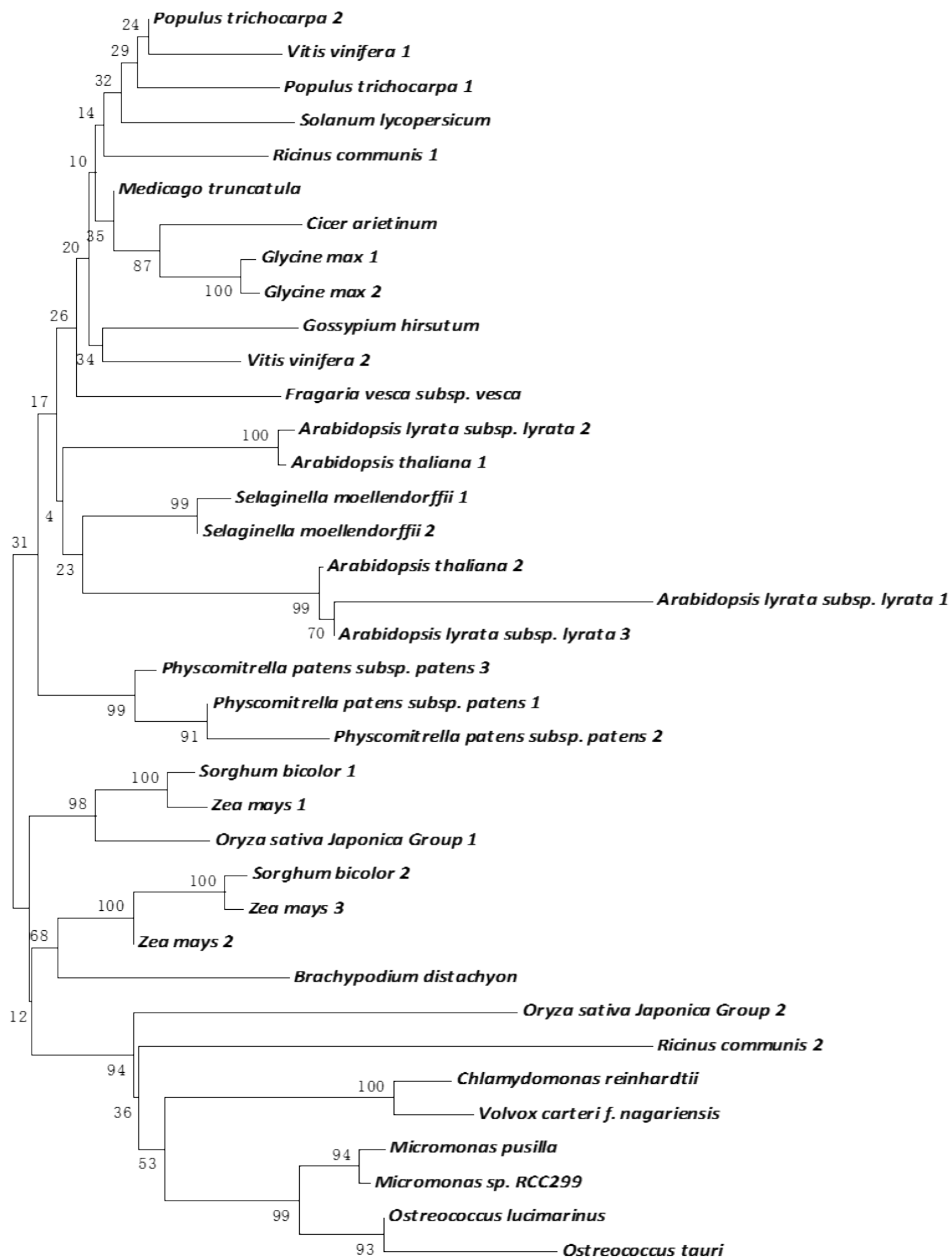

0.05

Supplement: Supplementary file 1 [file plants-12-03437-s001.zip › Figure S3.pdf]

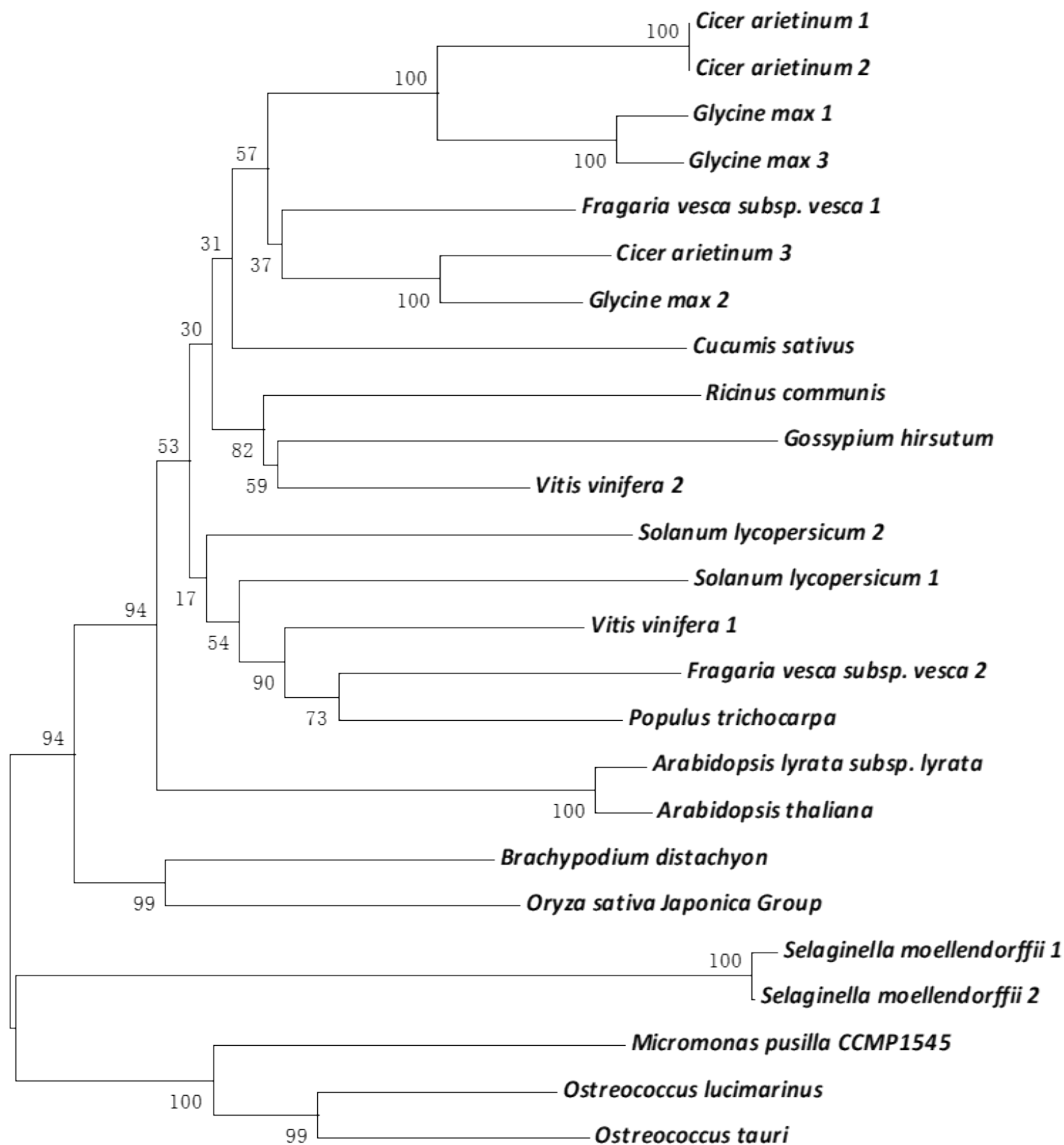

0.05

Supplement: Supplementary file 1 [file plants-12-03437-s001.zip › Figure S4.pdf]
